# The detection of canine anti-sperm antibody following parenteral immunization of bitches against homogenized whole sperm

**DOI:** 10.1186/s12610-020-0100-z

**Published:** 2020-01-23

**Authors:** Atefeh Esmailnejad, Behrooz Nikahval, Asghar Mogheiseh, Romina Karampour, Sanaz Karami

**Affiliations:** 1 Department of Pathobiology, School of Veterinary Medicine, Shiraz University, P.O.Box: 7144169155, Shiraz, Fars Iran; 2 Department of Clinical Sciences, School of Veterinary Medicine, Shiraz University, Shiraz, Fars Iran

**Keywords:** Anti-sperm antibody, Dog, Immunocontraception, Infertility, Sperm, Anticorps anti-sperme, Chien, immunocontraception, Infertilité, Sperme

## Abstract

**Backgrounds:**

The development of a canine-specific method of immunocontraception is one of the non-invasive controlling strategies for humanely decreasing the dog population. This study was aimed to investigate the potential of whole sperm in stimulating the immune system and producing specific anti-sperm antibodies (ASAs) in female dogs. Mature, mixed-breed bitches were subcutaneously immunized with high (200 × 10^6^ cells/mL) and low (100 × 10^6^ cells/mL) doses of sperm vaccine, emulsified with Freund’s adjuvants. Booster immunizations were given at weeks 1, 2, 4, and 6, and serum samples were collected at days 0, 14, 28, 42, 63, and 84 prior to each immunization. Reproductive tract samples, including vaginal and uterine lavages, were also collected by flushing each section with sterile PBS at the end of the experiment. Canine anti-sperm antibody titer and specificity in sera and genital secretions were measured using an enzyme-linked immunosorbent assay technique.

**Results:**

Specific anti-sperm antibodies were detected in the serum of both high and low dose groups and were significantly higher than those observed in the controls. A high dose of sperm induced elevated immune responses over the low dose antigen. Immunization with a high dose of sperm increased the level of ASAs in the uterine secretions and vaginal secretions significantly. Higher ASAs were observed to have transduced to the uterine lumen compared to the vagina.

**Conclusions:**

Based on the results obtained in this study, parenteral immunization with whole sperm can induce a high level of specific antibodies in the serum and genital secretions of female dogs and the response would be dose-dependent.

## Résumé

**Contexte:**

Le développement d’une méthode d’immunocontraception spécifique à la race canine constitue l’une des stratégies non invasives pour réduire humainement la population canine. Cette étude a pour objectif d’évaluer le potentiel du sperme entier à stimuler le système immunitaire des femelles et à produire des anticorps anti-sperme spécifiques (ASA) chez ces dernières. Des chiennes matures de race croisée sont immunisées par voir sous-cutanée avec soit de fortes doses (200 millions de cellules/mL) soit de faibles doses (100 millions de cellules/mL) de vaccin constitué de sperme entier émulsifié avec des adjuvants de Freund. Des vaccinations de rappel sont faites aux semaines 1, 2, 4 et 6, et des échantillons sanguins sont prélevés aux jours 0, 14, 28, 42, 63 et 84 avant chaque immunisation. Des échantillons de l’appareil reproducteur, incluant des lavages vaginaux et utérins, sont recueillis par flushing de chaque section avec du PBS stérile à la fin de l’expérimentation. Le titre et la spécificité des anticorps anti-sperme entier canin dans le sérum et les sécrétions génitales ont été mesurés par la technique de dosage ELISA.

**Résultats:**

Des anticorps anti-sperme entier spécifiques ont été détectés dans le sérum des femelles immunisées tant avec de faibles que de fortes doses, et de façon significativement plus élevé que chez le groupe témoin. Une dose forte de sperme entier induit des réponses immunitaires élevées par rapport à l'antigène à faible dose. L’immunisation avec une forte dose de sperme entier augmente de façon significative le niveau d’ASA dans les sécrétions utérines et dans les sécrétions vaginales. On a observé que les ASA ont plus été transduits vers la lumière utérine que vers la lumière vaginale.

**Conclusions:**

Basée sur les résultats obtenus dans la présente étude, l’immunisation parentérale par du sperme entier peut induire un taux élevé d’anticorps spécifiques dans le sérum et le sécrétions génitales de chiennes ; et la réponse serait dose-dépendante.

## Background

Available products for controlling fertility in dog population are limited to the synthetic hormonal preparations that prevent estrus and proestrus. Such products should be administered daily over a prolonged period of time to be effective [1]. Surgical spaying is invasive and also associated with postoperative side effects. One of the proposed non-invasive strategies for decreasing the stray dog populations is the immunologically-based fertility controlling method (immunocontraception) which reduces the breeding success in dogs. Immunocontraception induces infertility by stimulating the immune system to produce specific antibodies against key reproductive antigens and preventing fertilization [2, 3]. Naturally occurring anti-sperm antibodies (ASAs) in the serum and genital fluids of immunological infertile couples reflect the potential of ASA as an immunocontraceptive target [4–6].

Male germ cells, including spermatocytes and spermatozoa, are developed at puberty, long after the neonatal period and when self-tolerance mechanisms are established. Tight junctions in blood-testis barrier isolate these cells and prevent antibodies and immunocompetent cells from entering the lumen of seminiferous tubules. Therefore, sperm cells express antigens that have not been encountered by the immune system previously and act as autoantigens that elicit the immune responses in both males and females [7–10]. The role of immune responses against spermatozoa as a cause of infertility has been demonstrated in humans and several animal species including mice [11, 12], rats [13, 14], rabbits [15], dogs [16], possums [17], guinea pigs [18], foxes [19, 20], and cattle [21].

In cattle, semen treatment with immune sera prior to the insemination resulted in fertilization failure and early embryonic mortality. Anti-fertility effects of ASAs, including low pregnancy rate and high incidence of delayed return to the estrus, have also been demonstrated in heifers immunized with semen and bred artificially [21]. Although the role of anti-sperm antibodies in controlling the fertility has been verified in different animal species, there is no information regarding immunologic infertility due to the canine anti-sperm antibodies in bitches.

In the present study, the ability of the whole sperm in stimulating the immune system and producing some specific anti-sperm antibodies was investigated in female dogs. Canine anti-sperm antibody titer and specificity were evaluated in the serum samples and reproductive tract secretions. The ultimate goal of this research was to develop an immunocontraceptive vaccine candidate for the human control of the unowned or stray dog populations.

## Material and methods

### Statement of animal ethics

The study was approved by the Institutional Animal Care and Use Committee of Shiraz University (IACUC no: 4687/63). The recommendations of European Council Directive (2010/63/EU) of September 22, 2010, regarding the standards in the protection of animals used for experimental purposes, were also followed.

### Antigen preparation

Semen samples were collected by digital stimulation from 10 healthy, mature, male, mixed-breed dogs with reproductive known history. The quality of the semen was specified based on the sperm concentration (22 × 10^6^ sperm/kg body weight; SpermaCue device, Minitube International, Germany), sperm progressive motility (greater than 70% progressive motility), and sperm morphology (greater than 70% morphologically normal sperm) [22]. The semen samples were pooled together and centrifuged at 1200×g for 10 min at room temperature to separate the sperm cells. The pellet was resuspended in 10 mL sterile phosphate buffered saline (PBS) and the procedure was repeated three more times. Finally, the washed sperm cells were resuspended in sterile PBS at a concentration of 200 × 10^6^ and 100 × 10^6^ cells/mL and stored at − 20 °C until immunization.

### Immunization of dogs

A total of 15 healthy, mature, female, mixed-breed dogs with reproductive known history, with an average weight of 15–20 kg and age of 10 months were selected and divided into 3 equal groups (*n* = 5). All dogs were owned and kept by Shiraz University School of Veterinary Medicine. Anti-parasitic treatment was performed during the first 2 weeks of preparation by using praziquantel (10 mg/kg) and mebendazole (22 mg/kg). All dogs received 300 g/dog/day of commercial dog feed (Nutri® dry dog food; Behintash Co. Iran) and water was provided ad libitum. All dogs were evaluated using ultrasonography to determine stage of cycle and confirm non-pregnancy status.

The sperm vaccine was prepared by emulsifying one mL of whole sperm suspension with an equal volume of Freund’s complete adjuvant (CFA) (Sigma Aldrich, St Louis, MO, USA) in the first immunization and injected subcutaneously at 4 sites across the shoulder area. For the high dose, the dogs received 2 ml of vaccine containing 200 million sperm (which is 100 million sperm/ml) and for the low dose, the dogs received 100 million sperm/ml. Booster immunizations were given at weeks 1, 2, 4, and 6 replacing Freund’s complete adjuvant with Freund’s incomplete adjuvant (IFA; Sigma Aldrich, St. Louis, MO, USA) [23]. The control group (group 3) was administered with 1 mL of sterile PBS emulsified with 1 mL adjuvant in all immunizations.

### Sampling protocols

Blood samples were collected from jugular vein prior to immunizations at days 0, 14, 28, 42, 63, and 84. The serum samples were separated using centrifugation 750×g for 10 min and stored at − 20 °C before anti-sperm antibody analysis. At the end of the 12th week, all the dogs underwent the elective ovariohysterectomy (OHE) surgery and their ovaries and uterus were collected. The status of animals’ reproductive cycle and the presence of follicles and corpora lutea were recorded for all dogs. Finally, the samples of vaginal and uterine secretions were collected by flushing each section with 5 mL sterile PBS. The lavages were centrifuged and the supernatants were stored at − 20 °C to be used later in the analysis of antibodies.

### Assessment of anti-sperm antibodies

Anti-sperm antibody production and specificity in serum samples and the reproductive tract secretions were measured using an enzyme-linked immunosorbent assay (ELISA) technique. IgG antibodies were detected using a commercial indirect ELISA kit (Crystal Day Biotech, Shanghai, China. Cat. No. ED0299Ca), according to the manufacturer’s instructions. All the samples were run in duplicate. The inter-assay CV was < 10% and the intra-assay CV was < 12%. Microtiter plates were pre-coated with whole spermatozoa and blocked with bovine serum albumin. 50 μl of diluted serum and reproductive tract secretions (1:5) along with the negative and positive controls were added to the plate and incubated for 30 min at 37 °C. The plates were washed 5 times with PBS-Tween and incubated with 50 μl horseradish peroxidase-conjugated anti-canine IgG for 30 min at 37 °C. The plates were washed five additional times and color reaction was initiated by adding 50 μl tetramethylbenzidine substrate to the plates, incubated at 37 °C for 10 min. At the end of the experiment, the optical density (OD) of each sample was determined at 450 nm with a microtiter plate reader (Immunoskan BDSL, Thermo Lab. Systems, Finland) and used for comparison of anti-sperm antibody titers between groups [24].

### Statistical analysis

All data were expressed as means ± standard error of mean (SEM). The data were analyzed statistically by two-way analysis of variance (ANOVA) and Tukey’s multiple comparisons test to determine the degree of significance for ASAs titer between the control and experimental groups and also between the two experimental groups at different sampling time points. One-way ANOVA was also used to compare the uterine and vaginal ASAs levels in each group. The data were analyzed using GraphPad Prism software, version 6 for Windows, and the probability of *P* < 0.05 was considered statistically significant.

## Results

### Serum anti-sperm antibodies

Anti-sperm antibodies were detected in the serum of both high and low dose groups, but not in the sera of adjuvant-treated controls, a finding which confirmed the immunization protocol (*P* < 0.001). At day 0 and before the immunization, all the groups showed no antibody titer and their optical density was the same as the kit’s negative control (OD ~ 0.7).

**Fig. 1:**
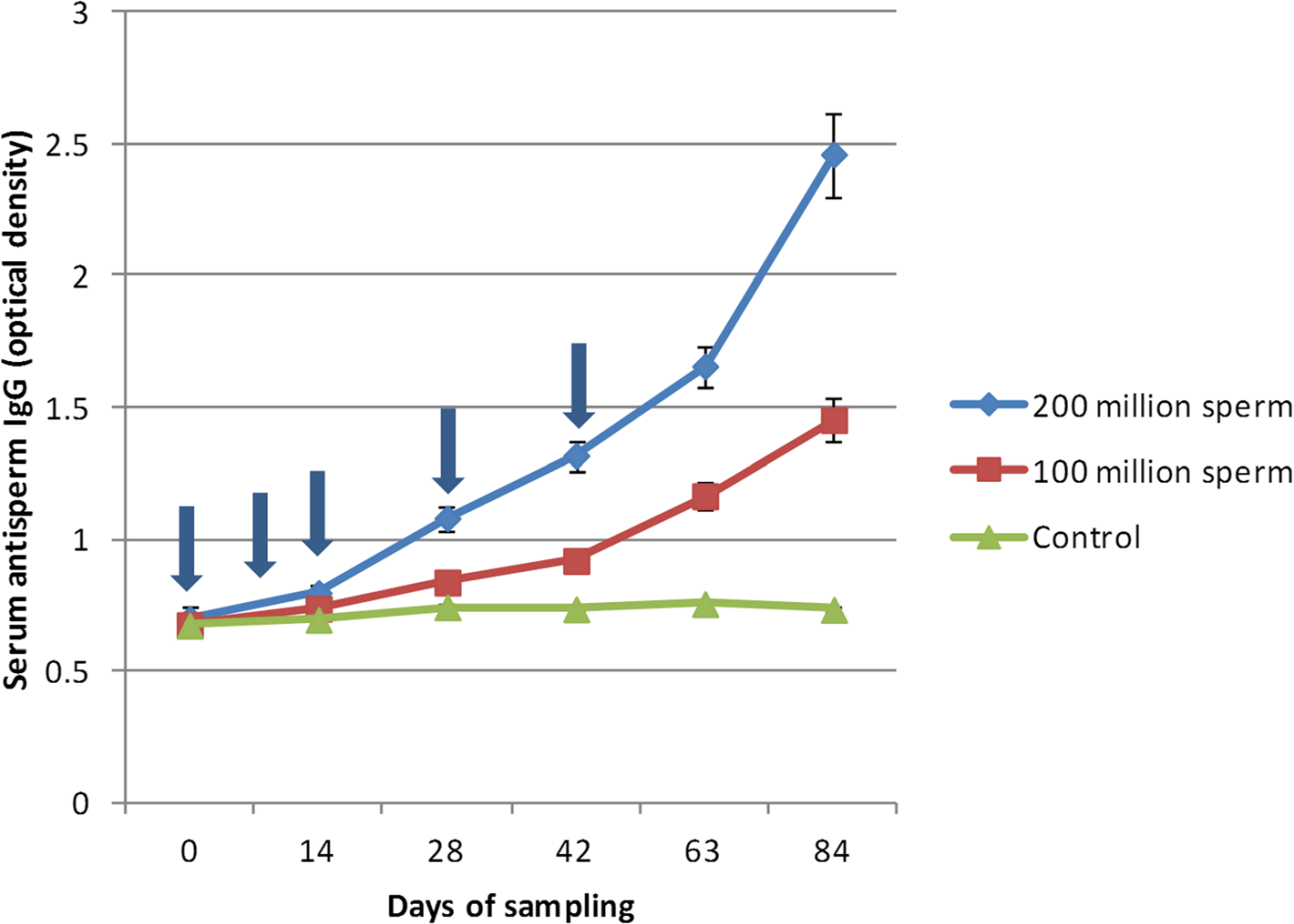
Changes of serum anti-sperm IgG during the immunization of bitches with a high (200 × 10^6^) and low (100 × 10^6^) dose of sperm. The arrows indicate times of subcutaneous injection of whole sperm vaccine

In the case of the high dose group, antibody response started to increase significantly following the third immunization (day 14). At day 28, the serum anti-sperm antibody level was significantly higher compared to that of the control (*P* = 0.0005). The ascending trend of ASAs production continued steadily in this group until the end of the experiment (day 84) (Fig. 1).

In the low-dose group, the production of the antibody began with a delay and a significant difference was observed in the titer compared with that of the control group at day 63, following the fifth immunization (*P* < 0.0001) (Fig. 1).

Comparing the antibody titers between high and low dose groups, the level of antibody was significantly higher in the high dose group from day 28 (*P* = 0.01) and remained higher until the end of the study (*P* < 0.0001).

Serum antibody titers were also compared in each group between different sampling time points (days 0, 14, 28, 42, 63, and 84). In the high dose group, the level of the antibody was significantly different between all the time points, except day 0 vs. day 14 (*P* = 0.8). In the low-dose group, the differences between days 0 vs. 14 (*P* = 0.9), 0 vs. 28 (*P* = 0.3), 14 vs. 28(*P* = 0.8), 14 vs. 42 (*P* = 0.2), and 28 vs. 42 (*P* = 0.9) were not statistically significant. The comparison of other sampling periods between day 0 vs. 42, 63 and 84; day 14 vs. 63 and 84; day 28 vs. 63 and 84; day 42 vs. 63 and 84; day 63 vs. 84 showed significant differences (*P* < 0.0001).

### Vaginal and uterine anti-sperm antibodies

At the end of the experiment, all the dogs were anestrus with no presence of dominant follicle (≥ 4 mm) or active corpora lutea. The immunization with a high dose of sperm increased the anti-sperm antibody titers in the vaginal secretions (*P* = 0.0004). The vaginal titer of ASAs was not significantly different between either the high and low dose groups (*P* = 0.052 or the low-dose and control (*P* = 0.1). The antibody response was also detected in the uterine secretions of both immunized groups although it was statistically different only in the high dose group compared to the control (*P* = 0.0003) (Table 1).

**Table 1:** A comparison of anti-sperm IgG among high (200 × 10^6^ sperm) dose, low (100 × 10^6^ sperm) dose and the control and between lavage samplings of vagina and uterus in bitches immunized with sperm

	Vagina	Uterus
High dose group (n = 5)	0.874 ± 0.03^a*^	1.25 ± 0.05^a*^
Low dose group (n = 5)	0.778 ± 0.01^ab^	0.996 ± 0.09^ab^
Control group (n = 5)	0.696 ± 0.01^b^	0.724 ± 0.01^b^

High dose = 200 × 10^6^ sperm; Low dose = 100 × 10^6^ sperm

*Indicates significant difference in each row

^ab^Superscript different letters indicate significant difference in each column

Also, the comparison of anti-sperm antibody titers between the vaginal and uterine secretions indicated a significantly higher transduction of antibodies to the uterine secretions in the high dose group (*P* = 0.0002). In the low-dose group, although the titer of ASAs was higher in the uterus, the difference was not significant (*P* = 0.057) (Table 1).

## Discussion

Anti-sperm antibodies interfere with fertilization through multiple mechanisms at different levels. The ASAs induced sperm agglutination that could impair the sperm motility [7, 23]. Antibodies in the reproductive tract secretions can cause sperm agglutination and immobilization, leading to a reduction in the number of sperms that reach the uterine tube, where the fertilization occurs [17]. Sperm penetration and migration through cervical mucus are also inhibited by the ASAs. Sperm coated by ASAs are easily targeted by neutrophils and macrophages for phagocytosis and destruction [25, 26]. Moreover, the ASAs may prevent the sperm from binding to the zona pellucida and impair the acrosome reaction [27]. The anti-fertility role of ASAs has also been reported after fertilization, through their interference with the blastocyst formation or embryo development [17, 28].

The identification of potential contraceptive immunogens specific to the reproductive process is a high priority in the development of immunocontraceptive controlling strategies for dog populations. The application of sperm cells as a candidate of contraceptive vaccine is contingent on raising a high titer of specific antibodies in the serum and genital tract that are capable of interfering with the fertilization process [23]. In this study, the parenteral immunization against whole sperm resulted in significant production of specific anti-sperm antibodies in the serum and reproductive tract secretions of female dogs. Immunization with a high dose of sperm (200 × 10^6^ cells/mL) resulted in a significantly higher antibody level both in the serum and genital tract fluids. Sperm immunogenicity and ASA production depend on different factors including the nature of the antigen, dose of the antigen, type of the adjuvant, route of administration, immunization protocol, and animal species [4]. The results of this study showed that immunization with lower doses of sperm (such as 100 × 10^6^ cells/mL) could be associated with a longer antibody production lag phase and also lower titer of ASAs in bitches. However, whether this antibody titer could induce infertility was not evaluated.

Anti-sperm antibodies occur spontaneously in humans and different species of animals [29–32] and can be associated with infertility [33–35]. Parenteral immunization of female brushtail possums with whole sperm reduced the pregnancy rate up to 80%, through the formation of ASAs in the serum and vaginal secretions [17]. Whole sperm administration via intramuscular (IM), subcutaneous (SC), and intranasal (IN) inoculations also caused ASAs formation in BALB/c mice serum, and the SC immunization was more efficient than the IM and IN procedures. However, ASAs were not detected in the vaginal washes in any experimental group [4]. Evidence for production of ASAs in bitches is limited to one study that aimed to introduce a reliable technique for assessing ASAs in dogs. Anti-sperm antibodies were detected in the serum samples of male and female dogs using indirect immunofluorescence and gelatin agglutination techniques, following immunization with sonicated whole sperm [16]. Contrary to the whole sperm immunogenicity findings, some investigations have indicated that most of the sperm antigens are surface epitopes that are likely to be shared with various somatic cells [8, 23, 36].

The importance of immunoglobulin classes in the reproductive tract immunity and the process by which antibodies reach the genital tract secretions are unknown in dogs. In this research, the parenteral immunization with a high dose of sperm significantly increased the anti-sperm antibody titers in the uterine and vaginal washes. Higher titers of ASAs were observed to have transduced to the uterine lumen in comparison to the vagina. Since the level of antibody in the genital secretions is influenced by the stage of the reproductive cycle [37, 38], vaginal and uterus fluids were collected when all the dogs were anestrus and the individual variation in the reproductive status was minimal. Studies on eutherian species indicated that the antibodies in the genital secretions may have been secreted locally or originated from the parenteral immune responses, later entering the lumen of uterus or vagina as a serum transudate [17, 38, 39]. A high degree of infertility following the parenteral immunization of possum species against whole sperm proposed that parenteral IgG might have a critical role in the reduced breeding of these animals [17]. Bouvet et al. [37] also investigated the possible induction of specific antibodies in the vaginal secretions following parenteral immunization in humans. The comparison between serum and vaginal flush ASA titers showed no differences in terms of specific activity or level of avidity. These results demonstrated that parenteral immunization could induce a serum-derived IgG release into the uterine and vaginal lumen. This method could reinforce or even replace the local vaccines [37]. Furthermore, the high titer of serum anti-sperm IgG and the increased level of IgG in the reproductive tract secretions of the high dose group in the current study suggest that ASAs could have been diffused from serum into the mucosal secretions.

In conclusion, the parenteral immunization with whole sperm can induce a high level of specific anti-sperm antibodies in the serum and genital secretions of female dogs. However, these results are dose-dependent and low-dose vaccines may need more extended immunization protocol, with longer intervals in dog populations. The fertility tests should be performed following parenteral immunization with whole sperm to confirm its validity as a contraceptive method for dogs.

## Data Availability

Data and materials are presented in the materials and methods section.

## Abbreviations

ANOVA: Analysis of variance
ASAs: Anti-sperm antibodies
CFA: Freund’s complete adjuvant
ELISA: Enzyme-linked immunosorbent assay
IM: Intramuscular
IN: Intranasal
OD: Optical density
OHE: Ovariohysterectomy
PBS: Phosphate buffered saline;
SC: Subcutaneous
SEM: Standard error of mean
